# Prescriptions

**Published:** 1896-08

**Authors:** 


### WHOOPING COUGH.

Dr. Murrell orders a mixture containing

℞. Oil of amber .......10 minims.
Powd. gum acacia.. 1 drachm.
Syr. of orange flower 2 drachms.
Oil of anise.... ....3 minims.
Water...... ... ..to 1 ounce.

For a linement the following formula is useful:

℞. Oil of amber........6 drachms.
Oil of rosemary....1 drachm.
Oil of origanum.....1 drachm.
Oil of turpentine......1 ounce.
Linseed-oil....... to 4 ounces.

—*Pediatrics.*

### SICK HEADACHE.

Critzman administers every two hours a capsule containing

℞. Sparteine sulphate...0.3 grain.
Caffein citrate .....1.5 grains.
Antipyrin...........8.0 grains.

M. Sig.: Four of these capsules are to be given, even though the pain may have completely disappeared.

If there is gastric intolerance, which frequently occurs, this mixture may be given in the form of an enema.—*New York Medical Journal.*

CHOREA.

℞. Sodium, salicylate.. ...... ʒii.
Syrup wintergreen.......... ℥i.
Wintergreen water, aa .... ℥ii.
M. Sig.: Twenty drops every hour for six hours, and thirty drops every two hours thereafter.—*Pediatrics.*

LOCAL ANESTHETIC.

Parson's Local Anesthetic is composed of

Chloroform............12 parts
Tincture of aconite....12 parts
Tincture of capsicum. . 4 parts
Tincture of pyrethrum 2 parts
Oil of cloves............ 2 parts
Gum camphor.......... 2 parts

Dissolve camphor in chloroform add oil of cloves, and lastly the tinctures.

ACUTE CYSTITIS.

℞. Potass. citratis..........dr. iv.
Sp. chloroformi.........oz. ijss.
Tr. digitalis..........drops 80.
Infus. buchu.........ad. oz. viii
M. Sig.: Two tablespoonfuls three or four times daily (Fothergill). The following suppository may be inserted high up in the rectum:
℞. Iodoformi................gr. 1.
Ext. hyoscyam..............gr. 1.
Oil theobromæ..........gr. xiv.
M. et. ft. sup. j.

